# Supplementary material for: Causal interplay between lactose intolerance and gut microbiota: a combined bidirectional Mendelian randomization and in vivo validation study
Source: Front Nutr. 2026 Jun 1;13:1803337. doi: 10.3389/fnut.2026.1803337 (PMC13265576; doi:10.3389/fnut.2026.1803337)
Supplement: Supplementary file 1 [file Data_Sheet_1.zip › supplementary materials/Forward/leave one out cross validation/ebi-a-GCST90027643.finngen_R12_E4_LACTONAS.pdf]

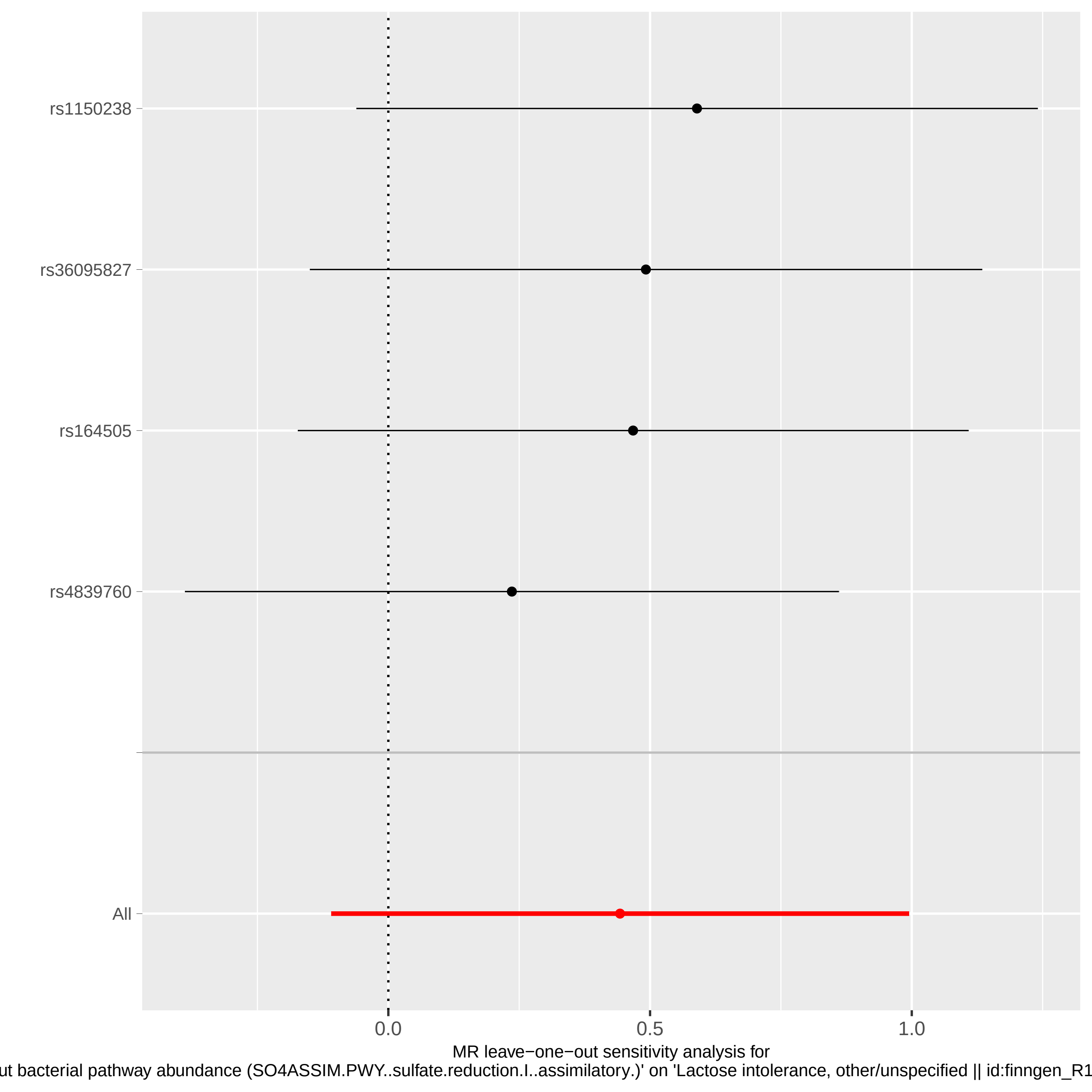

rs1150238
rs36095827
rs164505
rs4839760
All
0.0
0.5
1.0
MR leave−one−out sensitivity analysis for
ut bacterial pathway abundance (SO4ASSIM.PWY..sulfate.reduction.I..assimilatory.)' on 'Lactose intolerance, other/unspecified || id:finngen_R1
